# Proteome-Wide Alterations of Asymmetric Arginine Dimethylation Associated With Pancreatic Ductal Adenocarcinoma Pathogenesis

**DOI:** 10.3389/fcell.2020.545934

**Published:** 2020-12-03

**Authors:** Meijin Wei, Chaochao Tan, Zhouqin Tang, Yingying Lian, Ying Huang, Yi Chen, Congwei Chen, Wen Zhou, Tao Cai, Jiliang Hu

**Affiliations:** ^1^School of Pharmaceutical Sciences, Guangzhou University of Chinese Medicine, Guangzhou, China; ^2^Department of Clinical Laboratory, Hunan Provincial People’s Hospital, The First Affiliated Hospital of Hunan Normal University, Changsha, China; ^3^Translational Medicine Research Institute, Hunan Provincial People’s Hospital, The First Affiliated Hospital of Hunan Normal University, Changsha, China; ^4^Department of Emergency, Hunan Provincial People’s Hospital, The First Affiliated Hospital of Hunan Normal University, Changsha, China; ^5^Department of Neurosurgery, The Third Xiangya Hospital, Central South University, Changsha, China

**Keywords:** protein methylation, asymmetric dimethylarginine, pancreatic ductal adenocarcinoma, PRMT4, label-free quantitative proteomics

## Abstract

Arginine methylation catalyzed by protein arginine methyltransferases (PRMTs) performs essential roles in regulating cancer initiation and progression, but its implication in pancreatic ductal adenocarcinoma (PDAC) requires further elucidation. In this study, asymmetric dimethylarginine (ADMA)-containing peptides in PDAC cell line PANC-1 were identified by label-free quantitative proteomics combined with affinity purification, using human non-cancerous pancreatic ductal epithelium cell line HPDE6c7 as the control. In total, 289 ADMA sites in 201 proteins were identified in HPDE6c7 and PANC-1 cells, including 82 sites with lower dimethylation and 37 sites with higher dimethylation in PANC-1 cells compared with HPDE6c7 cells. These ADMA-containing peptides demonstrated significant enrichment of glycine and proline residues in both cell lines. Importantly, leucine residues were significantly enriched in ADMA-containing peptides identified only in HPDE6c7 cells or showing lower dimethylation in PANC-1 cells. ADMA-containing proteins were significantly enriched in multiple biological processes and signaling cascades associated with cancer development, such as spliceosome machinery, the Wnt/β-catenin, Hedgehog, tumor growth factor beta (TGF-β), and mitogen-activated protein kinase (MAPK) signaling pathways. Moreover, PDAC cell lines with enhanced cell viability showed lower PRMT4 protein abundance and global ADMA-containing protein levels compared with HPDE6c7. PRMT4 overexpression partially recovered ADMA-containing protein levels and repressed viability in PANC-1 cells. These results revealed significantly altered ADMA-containing protein profiles in human pancreatic carcinoma cells, which provided a basis for elucidating the pathogenic roles of PRMT-mediated protein methylation in pancreatic cancer.

## Introduction

Pancreatic cancer is a common malignant disorder with rapid progression and poor prognosis and remains one of the leading causes of cancer related deaths worldwide (Chen et al., 2016; Siegel et al., 2018). Pancreatic ductal adenocarcinoma (PDAC) is a major pancreatic cancer subtype, accounting for more than 85% of global pancreatic cancer cases, with a 5-year survival rate of less than 5% (Ryan et al., 2014; Siegel et al., 2018). PDAC pathogenesis is driven by multiple genetic alterations such as the activating mutation of KRAS (v-Ki-ras2 Kirsten rat sarcoma viral oncogene homolog) (Ryan et al., 2014; Buscail et al., 2020). However, current therapeutic regimens targeting KRAS have failed to lower PDAC mortality and improve prognosis, partially due to limited effectiveness (Iovanna and Dusetti, 2017; Buscail et al., 2020). In recent years, post-translational modifications (PTMs) have emerged as essential regulators of PDAC initiation and progression (Roth et al., 2017; Tan et al., 2019; Pan et al., 2020). The identification and functional investigation of protein modifications associated with PDAC pathogenesis could provide alternative targets for pancreatic cancer diagnosis and treatment.

Arginine methylation refers to the addition of methyl groups onto guanidino groups localized on the side chains of protein arginine (Arg) residues, and was first discovered as a protein modification type in 1971 (Bedford and Clarke, 2009; Blanc and Richard, 2017; Guccione and Richard, 2019; Jarrold and Davies, 2019). Arginine methylation is catalyzed by the protein arginine methyltransferases (PRMTs), resulting in the formation of monomethylarginine (MMA), asymmetrical dimethylarginine (ADMA), or symmetrical dimethylarginine (SDMA) (Guccione and Richard, 2019; Jarrold and Davies, 2019). Methylation increases the hydrophobicity and bulkiness of arginine residues in modified proteins and consequently interferes with their interactions with other proteins or nucleic acid partners (Shishkova et al., 2017). Arginine methylation is closely involved in various biological processes through affecting gene transcription, pre-mRNA splicing, protein translation, and synthesis (Guccione and Richard, 2019; Jarrold and Davies, 2019). Importantly, arginine methylation deregulation is also closely associated with cancer initiation, metastasis, and drug resistance (Yang and Bedford, 2013; Guccione and Richard, 2019; Jarrold and Davies, 2019). For instance, the asymmetrical dimethylation of histone H4 at position R3 (H4R3me2a) by PRMT1 mediates the epigenetic reprogramming and aberrant transcriptional regulation during the progression of acute myeloid leukemia (Cheung et al., 2016). Arginine methylation on histone tails have been established as key epigenetic events that drive cancer development and progression (Waldmann and Schneider, 2013; Jarrold and Davies, 2019).

Recent research has showed that arginine methylation in non-histone proteins also performs pivotal roles in cancer pathogenesis (Biggar and Shawn, 2015). For instance, PRMT4, alternatively known as co-activator-associated arginine methyltransferase 1 (CARM1), is overexpressed in breast cancer cells and regulates breast cancer progression and chemo-sensitivity through catalyzing the arginine methylation of multiple proteins such as pyruvate kinase M2 (PKM2) isoform, BAF155, and RNA polymerase II mediator complex subunit 12 (MED12) (Wang et al., 2014, 2015; Liu et al., 2017). However, the expression of PRMT4 protein is significantly suppressed in pancreatic cancer cells, resulting in reduced asymmetric arginine dimethylation of malate dehydrogenase 1 (MDH1) and enhanced non-canonical glutamine metabolism (Wang Y. P. et al., 2016). Moreover, asymmetric arginine dimethylation of other non-histone proteins such as Gli1 (glioma-associated oncogene homolog) and ATP-binding cassette subfamily G member 2 (ABCG2) were also implicated in pancreatic cancer pathogenesis, suggesting the important roles of asymmetric arginine dimethylation in PDAC (Wang Y. et al., 2016; Hsu et al., 2018). However, current knowledge of asymmetric arginine dimethylation in PDAC pathogenesis remains limited due to the lack of large-scale characterization of ADMA-containing proteins in pancreatic cancer cells (Pan et al., 2020).

Mass spectrometry-based proteomics combined with immunoaffinity purification has been successfully applied for profiling protein arginine methylation in *Trypanosoma brucei* (Fisk et al., 2013), *Plasmodium falciparum* (Zeeshan et al., 2017), *Toxoplasma gondii* (Yakubu et al., 2017), human renal epithelial cells (Sylvestersen et al., 2014), T cells (Geoghegan et al., 2015), breast and colon cancer cells (Guo et al., 2014; Shishkova et al., 2017). In this study, we performed a global characterization of ADMA-containing proteins in human pancreatic ductal epithelial cells and PDAC cells through label-free quantitative proteomics coupled with affinity purification, which laid a foundation for elucidating the roles of PRMT-mediated protein methylation in PDAC pathogenesis.

## Materials and Methods

### Cell Culture, Transfection, and Viability

The immortalized human pancreatic ductal epithelium cell line HPDE6c7 was obtained from the Kyushu University (Japan) and two PDAC cell lines PANC-1 and BxPC-3 were purchased from the Type Culture Collection of the Chinese Academy of Sciences (Shanghai, China). Short tandem repeat (STR) profiling was used to authenticate cell identity. Cells were cultured in DMEM (Dulbecco Modified Eagle Medium) containing 10% fetal bovine serum (Invitrogen) and penicillin/streptomycin at 37°C with 5% CO_2_. Human PRMT4 gene coding sequences containing HA-tag sequence at N-terminal were amplified by RT-PCR and ligated with the Lv-CMV-EGFP vector (Cyagen Biosciences, Suzhou, China), and the packaged recombinant lentivirus vectors were transfected into PANC-1 cells for stable expression of HA-PRMT4 proteins, as previously described (Wu et al., 2012). PANC-1 cells transfected with packaged Lv-CMV-EGFP vectors without ligation of PRMT4 gene coding sequences were used as the control group. Cell viabilities were measured by the CCK-8 (Cell Counting Kit-8; Dojindo, Japan) for three biological replicates as previously introduced (Tan et al., 2019).

### Protein Extraction and Quality Control

Total proteins were extracted from approximately 2 × 10^8^ cells at 80% confluency using a lysis buffer containing 100 mM HEPES (pH 8.0), 8 M urea, and 1% protease inhibitor cocktail (Sigma-Aldrich). After sonication and centrifuged at 20,000 × *g* for 15 min, the concentrations of proteins in the supernatant fraction were determined by the bicinchoninic acid (BCA) method. Protein quality evaluation was performed by Sodium dodecyl sulfate-polyacrylamide gel electrophoresis (SDS-PAGE) separation combined with Coomassie brilliant blue staining, and the numbers of protein groups were identified by a preliminary liquid chromatography-tandem mass spectrometry (LC-MS/MS) analysis.

### Tryptic Digestion

Total proteins (15 mg/group) were mixed with 1.25 M DTT (dithiothreitol; final concentration:10 mM) and incubated at 37°C for 30 min with gentle shaking, followed by incubation with 50 mM iodoacetamide (IAM) in darkness for 30 min at room temperature. Subsequently, proteins were digested in a solution with trypsin (Sigma-Aldrich) at 37°C for 17 h, as previously described (Hu et al., 2015). The resulting peptide solution was mixed with 0.1% trifluoroacetic acid (TFA), desalted by solid-phase extraction on a C18 cartridge column, and lyophilized for approximately 2 days to remove TFA, as previously described (Hu et al., 2015).

### ADMA-Containing Peptide Enrichment

The enrichment of ADMA-containing peptides was done using the PTMScan^®^ Asymmetric Di-Methyl Arginine Motif Kit (#13474; Cell Signaling Technology), following the manufacturer’s instructions. Briefly, peptide powders were resuspended with 1.4 mL pre-chilled IAP buffer and incubated with one vial of immunoaffinity beads for 1.5 h at 4°C. After being centrifuged at 2,000 × *g* for 30 s, the supernatant was removed, and immunoaffinity beads were washed three times with pre-chilled IAP buffer and washed three times with pre-chilled ddH_2_O. Subsequently, immunoaffinity beads were eluted twice with 40 μL 0.15% TFA by incubation at room temperature for 10 min. The resulting peptide solution was desalted on a C18-StageTips column and dried by lyophilization in a vacuum concentrator for mass spectrometry.

### LC-MS/MS

Asymmetric dimethylarginine-containing peptide powders were then dissolved in 5% acetonitrile containing 0.1% TFA, which were subjected to LC-MS/MS (liquid chromatography coupled with tandem mass spectrometry) using a Thermo Scientific Q Exactive MS system coupled online to an Easy-nLC 1000 instrument. Peptide solution was first loaded into the Thermo Scientific Acclaim PepMap100 loading column (100 μm × 20 mm, nanoViper C18) through the autosampler, followed by separation on a Thermo Scientific EASY column (75 μm × 250 mm) packed with C18-A2 particles (3 μm). Buffer A (0.1% FA) and Buffer B (84% acetonitrile and 0.1% FA) were used as the mobile phases. The flow rate was set to 300 nL/min using the following non-linear gradient: 5–8% buffer B, 10 min; 8–20% buffer B, 1 h; 20–30% buffer B, 7 min; 30–100% buffer B, 3 min; 100% buffer B, 12 min. Tandem mass spectrometry was performed in the positive ion mode, and precursor ions ranging from 300 to 1800 m/z were measured at 70,000 resolution (200 m/z) with an automatic gain control target of 10^6^ ions and a maximum injection time of 10 ms. Twenty precursor ions with the highest intensities from each full scan were selected for fragmentation by higher-energy C-trap dissociation (HCD) (normalized collision energy: 30 eV). MS2 spectra were acquired at a resolution of 17,500 at 200 m/z, and the isolation window was set to 2 m/z. All mass spectrometry data were deposited to the ProteomeXchange Consortium using the iProX partner repository (Ma et al., 2019) with the dataset identifier PXD017577^1^.

### Database Searching

Raw data from LC/MS/MS analysis were searched against the Uniprot proteome database released on 30/1/2018 (taxonomy: Homo sapiens) containing 20,244 canonical and isoform entries using the MaxQuant software (version: 1.6.0.16)^2^. The protease for protein digestion was set to trypsin and the maximum allowable mis-cleavages were set to two. A minimum peptide length of 7 amino acids and a maximum peptide mass of 4,600 Da were used for the database search. The mass tolerances for both precursor ions and fragment ions were 20 PPM. Cysteine carbamidomethylation was used as the fixed modification and variable modifications included N-terminal acetylation, methionine oxidation, and arginine dimethylation (28.0313 Da). A false discovery rate (FDR) of <0.01 was used for both peptide and protein identification. An Andromeda score of >40 and a localization probability of >0.75 were used for acceptance of peptide modification. Other parameters were set to default values.

### Quantitation and Bioinformatics

The differential levels of ADMA-containing peptides between the HPDE6c7 and PANC-1 cells were analyzed by Perseus software (version 1.6.6.0)^3^ following the manufacturer’s instructions (Tyanova et al., 2016). Briefly, the “Dimethyl (R)Sites.txt” file produced from database search was loaded to the Perseus platform, followed by filtering out contaminants and reverse (decoy) database hits. ADMA sites with a localization probability of <0.75 were excluded. The peptide intensities were logarithm-transformed using the formula “log2(x)” followed by median normalization. The Pearson correlation between biological replicates were >0.977 in the HPDE6c7 group and >0.785 in the PANC-1 group. After adding the missing data, we defined the significantly differential ADMA-containing peptides by the Student’s *t*-test (*P* < 0.05), which were then used for hierarchical clustering and PCA analysis. Consensus sequences of ADMA-containing peptides were predicted by the online pLogo platform^4^ as previously introduced (O’Shea et al., 2013). We analyzed functional categorization based on Gene ontology (GO) and Kyoto Encyclopedia of Genes and Genomes (KEGG) signaling pathways using the DAVID database (Huang da et al., 2009). The signaling pathway diagrams of ADMA-containing proteins were modified from the KEGG database^5^.

### Immunoblotting

Protein abundances and total ADMA-containing protein levels between HPDE6c7 and PANC-1 cells were detected by western blotting, as previously introduced (Hu et al., 2017). Primary antibodies targeting PRMT4 (#4438; Cell Signaling Technology), ADMA (#13522; Cell Signaling Technology), and β-actin (#ab8227; Abcam) were used in this study. At least three biological replicates were done for protein quantitation.

### Experimental Design and Statistical Rationale

HPDE6c7 and PANC-1 cells cultured under the same conditions were subjected to ADMA-containing peptide identification by label-free quantitative proteomics. Three biological replicates were performed in each cell line (*n* = 3). ADMA-containing peptides enriched from each sample were continuously analyzed by LC-MS/MS. All raw data were searched together against the Uniprot proteome database using the MaxQuant software. The FDR of <0.01 and Andromeda score of >40 were used as thresholds for ADMA-containing peptide and protein identification. Differentially methylated peptides were defined by a *P*-value of <0.05 from Student’s *t*-test using the Perseus software. Significant differences in other assays (*P* < 0.05) were evaluated by the Student’s *t*-test or analysis of variance (ANOVA) using the SPSS 20.0 software.

## Results

### Global Profiling of ADMA-Containing Proteins in HPDE6c7 and PANCI-1 Cells

**FIGURE 1:**
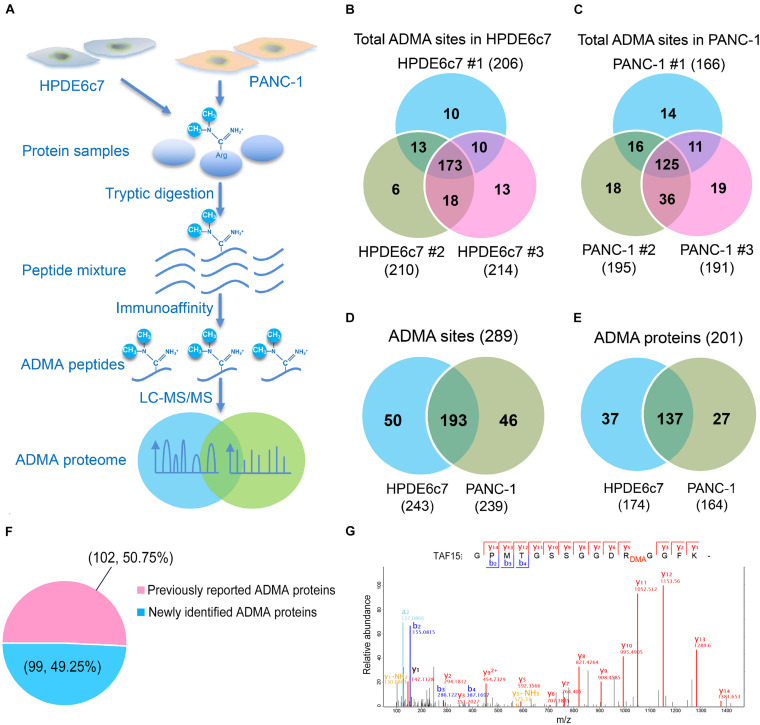
Mass spectrometric identification of ADMA-containing peptides in HPDE6c7 and PDAC cells. **(A)** A schematic illustration of proteomic characterization of ADMA-containing peptides in pancreatic cells. ADMA-containing peptides in PANC-1 cells were enriched by immunoaffinity and identified by LC-MS/MS, and human normal pancreatic ductal epithelium cell line HPDE6c7 was used as the control. **(B,C)** ADMA sites identified in HPDE6c7 and PANC-1 cells by three biological replicates. ADMA site numbers in HPDE6c7 **(B)** and PANC-1 **(C)** cells were shown in Venn diagrams. **(D)** Total numbers of ADMA sites in HPDE6c7 and PANC-1 cells. **(E)** Total numbers of ADMA-containing proteins in HPDE6c7 and PANC-1 cells. **(F)** Percentage of previously reported and newly identified ADMA-containing proteins. The numbers of previously reported or newly identified ADMA-containing proteins were shown in the brackets together with their percentages. **(G)** Mass spectrometric identification of TAF15 protein asymmetric dimethylation at Arg206 in PANC-1 cells.

For the profiling of asymmetric arginine dimethylation, ADMA-containing peptides in both HPDE6c7 and PANC-1 cells were identified by label-free quantitative proteomics following immunoaffinity purification (Figure 1A). In total, 289 ADMA sites were identified by three biological replicates, including 243 and 239 ADMA sites, which were identified in the HPDE6c7 and PANC-1 cells, respectively (Figures 1B,C and Supplementary Table 1). Among them, 193 ADMA sites were identified in both cell lines, while 50 and 46 ADMA sites were identified exclusively in HPDE6c7 and PDAC cells, respectively (Figure 1D). These ADMA-containing peptides were also mapped to a total of 201 methylated proteins, including 174 and 164 methylated proteins in HPDE6c7 and PDAC cells, respectively (Figure 1E and Supplementary Table 1). Among them, the asymmetric dimethylation of 50.75% (102/201) proteins were previously reported, validating the ADMA-containing protein dataset (Figure 1F and Supplementary Table 1). The previously reported asymmetric dimethylation of TATA-binding protein associated factor 15 (TAF15) protein at Arg206 (Jobert et al., 2009), was also detected in our proteomic analysis (Figure 1G). Moreover, 99 ADMA-containing proteins were newly identified in this study, and their possible roles in PDAC pathogenesis deserve further investigation in future.

### Differential Asymmetric Arginine Dimethylation in Pancreatic Cancer Cells

**FIGURE 2:**
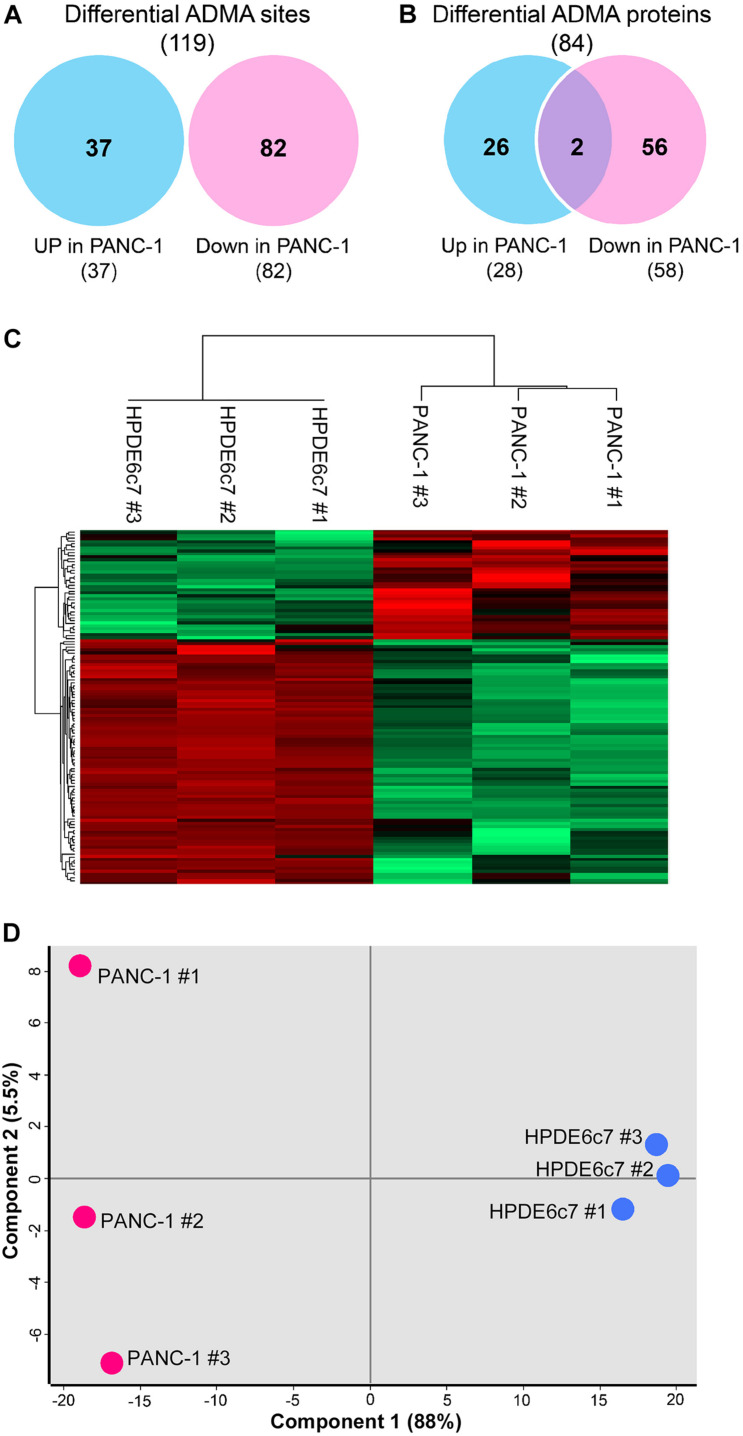
Significantly differential ADMA profiles between HPDE6c7 and PDAC cells. **(A,B)** Numbers of ADMA sites and ADMA-containing proteins with significantly differential dimethylation levels between HPDE6c7 and PANC-1 cells. Differentially dimethylated arginine sites were defined by *P* < 0.05 using the Perseus software and shown in Venn diagrams. Totally 119 ADMA sites showing differential dimethylation between HPDE6c7 and PANC-1 cells originate from these 193 overlapping sites shown in Figure 1D. **(C)** Hierarchical clustering of ADMA sites showing differential dimethylation between HPDE6c7 and PANC-1 cells. The higher and lower dimethylation levels are shown by red and green colors, respectively. **(D)** The PCA evaluation of differential asymmetric arginine dimethylation between HPDE6c7 and PANC-1 cells. Biological replicates of HPDE6c7 and PANC-1 cells are shown in blue and red circles, respectively. The proportions of the variance of component 1 and 2 are shown in the brackets.

Label-free quantitation of ADMA-containing peptides was subsequently performed using the Perseus software. We found that 119 of the total 193 ADMA sites identified in both the HPDE6c7 and PANC-1 cells, were differentially dimethylated between these two cell lines (Figure 2A and Supplementary Table 2). Among them, 82 ADMA sites showed lower dimethylation levels in PANC-1 cells compared with HPDE6c7 cells, which is much more than those showing higher dimethylation in PANC-1 cells (Figure 2A). The dimethylation of 58 proteins in PANC-1 cells was significantly lower than the HPDE6c7 cells, while only 28 proteins showed higher dimethylation in PANC-1 cells (Figure 2B). Our hierarchical clustering also demonstrated significantly differential arginine dimethylation between HPDE6c7 and PANC-1 cells, and the majority of differential ADMA-containing proteins showed lowered dimethylation levels in PANC-1 cells compared with HPDE6c7 cells (Figure 2C). Moreover, biological replicates of HPDE6c7 and PANC-1 cells were separated in the principal component analysis (PCA), based on differentially dimethylated proteins (Figure 2D). Together, these results show significantly differential ADMA-containing protein profiles between the HPDE6c7 and PANC-1 cells.

### Lower Leucine Enrichment Flanking ADMA Sites in PDAC Cells

**FIGURE 3:**
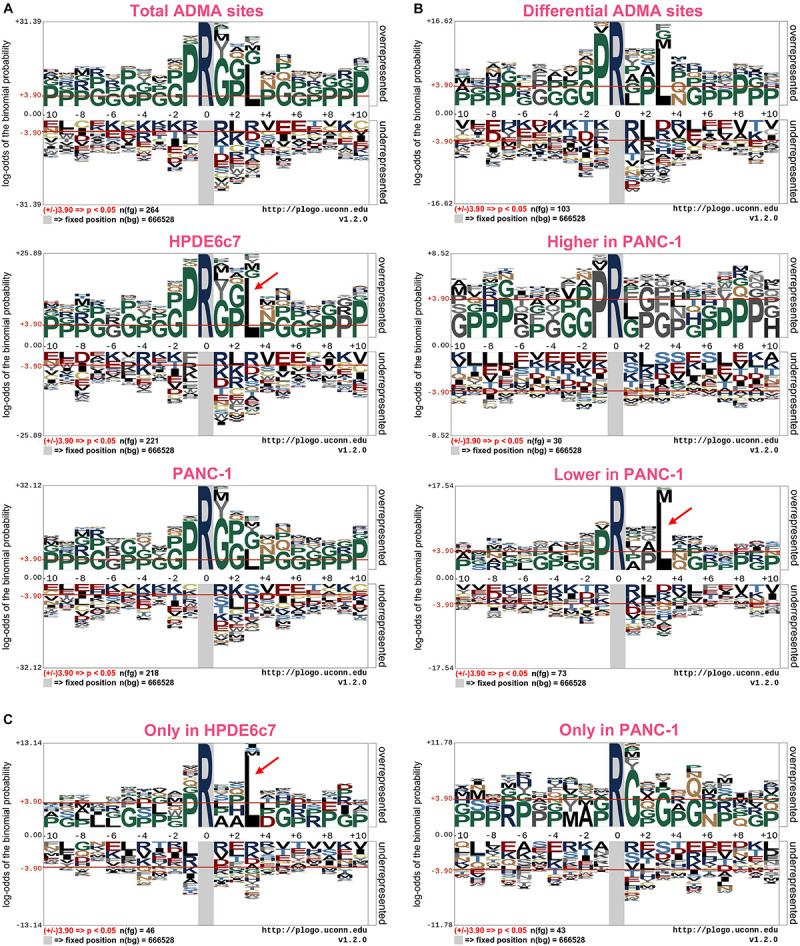
Consensus sequences of ADMA-containing peptides in PDAC cells. **(A)** Consensus sequences of a total of 351 ADMA-containing peptides identified in HPDE6c7 and PANC-1 cells. **(B)** Consensus sequences of ADMA-containing peptides differentially dimethylated between HPDE6c7 and PANC-1 cells. The enrichment was based on 119 ADMA-containing peptides with significantly differential dimethylation between HPDE6c7 and PANC-1 cells. **(C)** Consensus sequences of ADMA-containing peptides, detected only in HPDE6c7 or PANC-1 cells. The dimethylated arginine residues are indicated in dark blue. Frequencies of amino acid residues flanking ADMA sites were analyzed using the pLogo software, and significant enrichment was defined by *P* < 0.05. The significant overrepresentation of leucine is highlighted by red arrows.

Previous reports have revealed significant enrichment of proline and glycine residues in the vicinity of ADMA sites (Uhlmann et al., 2012; Fisk et al., 2013; Guo et al., 2014; Shishkova et al., 2017). With this large set of ADMA-containing peptides, we also characterized consensus sequences of asymmetric arginine dimethylation using the pLogo software. We showed that proline (P) at positions −1 and glycine at multiple positions were significantly overrepresented in ADMA-containing peptides in both HPDE6c7 and PANC-1 cells (Figure 3A and Supplementary Table 1). This observation is consistent with previous reports (Uhlmann et al., 2012; Fisk et al., 2013; Guo et al., 2014; Shishkova et al., 2017), suggesting unbiased ADMA-containing peptide identification in our proteomic analysis. More importantly, we showed that leucine residue at position +3 was preferentially present in ADMA-containing peptides, especially in those identified in HPDE6c7 cells and differentially dimethylated between HPDE6c7 and PANC-1 cells (Figures 3A,B and Supplementary Table 1). The frequency of leucine at position +3 was even higher than proline and glycine in ADMA-containing peptides with lower dimethylation in PANC-1 cells, but not in those with higher dimethylation in PANC-1 cells (Figure 3B and Supplementary Table 2). Consistently, the enrichment of leucine residues was observed in ADMA-containing peptides identified only in HPDE6c7 cells, other than those identified only in PANC-1 cells (Figure 3C and Supplementary Table 1). These results suggested the differential properties of ADMA-flanking residues between HPDE6c7 and PANC-1 cells.

### Functional Categorization of ADMA-Containing Proteins

**FIGURE 4:**
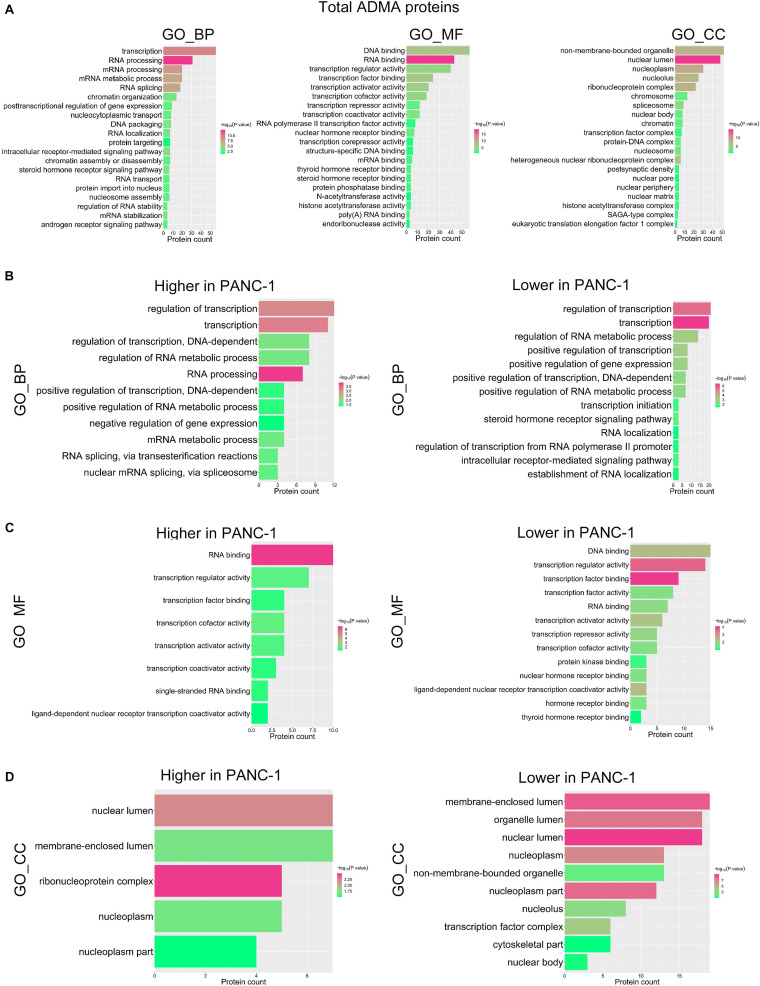
Functional categorization of ADMA-containing proteins in PDAC cells. **(A)** Functional annotation of total ADMA-containing proteins in HPDE6c7 and PANC-1 cells. The GO biological processes (BP), molecular functions (MF), and cellular components (CC) with significant enrichment of ADMA-containing proteins are presented as red and green bars (*P* < 0.05). **(B–D)** Functional categorization of ADMA-containing proteins showing significantly differential dimethylation between HPDE6c7 and PANC-1 cells. The classification of ADMA-containing proteins were based on GO biological processes **(B)**, molecular functions **(C)**, and cellular components **(D)**, respectively.

To explore the potential biological roles of asymmetric arginine dimethylation, we then performed a functional categorization of ADMA-containing proteins in HPDE6c7 and PANC-1 cells based on Gene Ontology (GO) terms. In general, these modified proteins were significantly enriched in several biological processes associated with RNA processing and gene transcription, especially mRNA processing and stability, RNA splicing, RNA localization and transport, chromatin organization, nucleosome assembly, and transcription initiation (Figures 4A,B). The separate categorization of ADMA-containing proteins in HPDE6c7 and PANC-1 cells disclosed enrichments in biological processes similar to the total ADMA-containing proteins (data not shown). In terms of molecular functions, these ADMA-containing proteins possessed pleiotropic activities as transcription factors, transcription activators or repressors, RNA polymerase II transcription factors, and DNA or mRNA binding molecules (Figures 4A,C). These ADMA-containing proteins were also significantly enriched in nuclear components such as nuclear lumen, nucleoplasm, nucleolus, chromosome, nuclear body, and spliceosome (Figures 4A,D). Furthermore, many ADMA-containing proteins were associated with signaling pathways mediated by intracellular receptors, steroid hormone receptors, and androgen receptors (Figure 4A), which was mainly observed in proteins with lower ADMA levels in PANC-1 cells compared with HPDE6c7 cells, but not in those showing higher ADMA levels in PANC-1 cells (Figure 4B).

### ADMA-Containing Proteins Enriched in Spliceosome and Cancer-Related Pathways

**FIGURE 5:**
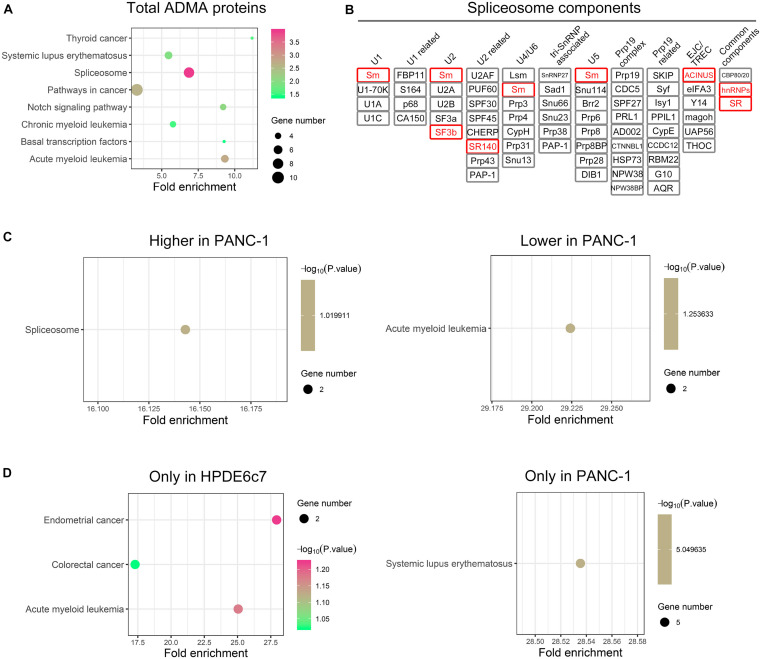
The enrichments of ADMA-containing proteins in KEGG pathways. **(A)** Signaling pathways with significant enrichment of total ADMA-containing proteins detected in HPDE6c7 and PANC-1 cells. The enrichments in KEGG signaling pathways were analyzed by searching against the Database for Annotation, Visualization, and Integrated Discovery (DAVID). **(B)** ADMA-containing spliceosome component proteins identified in HPDE6c7 and PANC-1 cells. ADMA-containing proteins identified in this study are shown in red rectangular. **(C)** The enrichment of ADMA-containing proteins with differential dimethylation between HPDE6c7 and PANC-1 cells in KEGG signaling pathways. **(D)** KEGG signaling pathways with enrichments of ADMA-containing proteins detected exclusively in HPDE6c7 or PANC-1 cells.

For more insights into the possible roles of asymmetric arginine dimethylation in PDAC, we further analyzed the enrichment of ADMA-containing proteins in KEGG signaling pathways. These ADMA-harboring proteins in HPDE6c7 and PANC-1 cells were significantly associated with spliceosome, basal transcription, systemic lupus erythematosus, Notch signaling, and multiple cancer pathways including acute myeloid leukemia, chronic myeloid leukemia, and thyroid cancer (Figure 5A and Supplementary Figures 1–7). Specifically, several spliceosome components were asymmetrically dimethylated including Sm, SF3b, SR140, ACINUS, hnRNPs, and SR (Figure 5B). Moreover, two ADMA-containing proteins showing higher dimethylation in PANC-1 cells were related to the spliceosome pathway, suggesting the involvement of RNA splicing regulation by asymmetric arginine dimethylation during PDAC development (Figure 5C).

**FIGURE 6:**
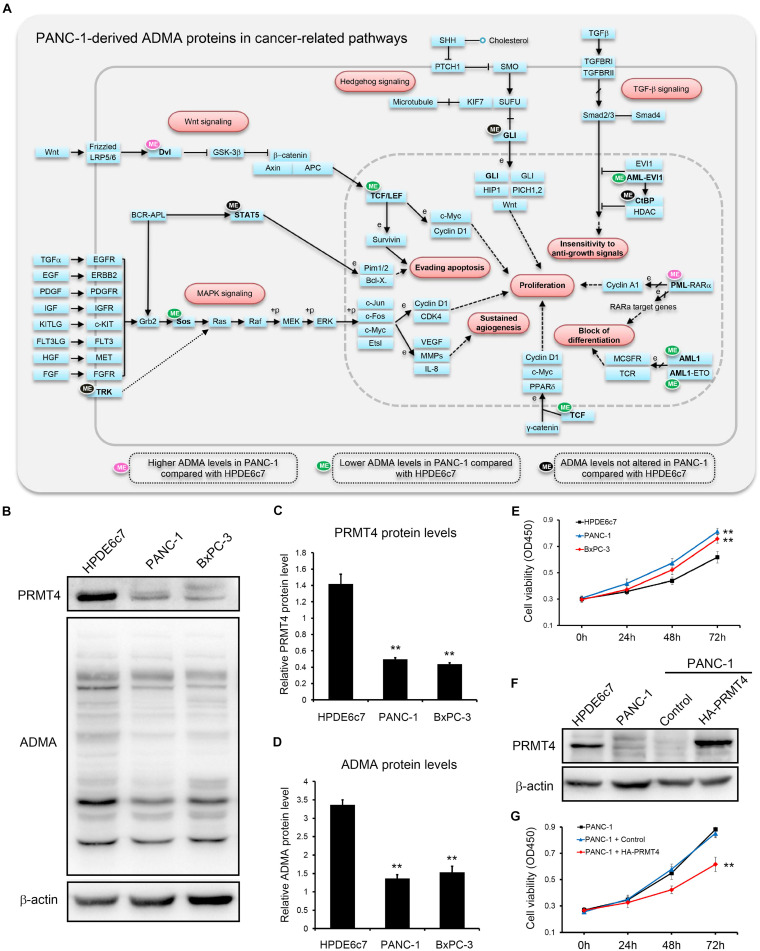
Extensive asymmetric arginine dimethylation associated with PDAC cell viability. **(A)** ADMA-containing proteins in major cancer-related signaling pathways. Total ADMA-containing proteins were searched against the KEGG pathway database (www.kegg.jp). ADMA-containing proteins showing higher, lower, or similar asymmetric arginine dimethylation levels in PANC-1 cells by proteomics are indicated by pink, green, and black ellipses, respectively, compared with HPDE6c7 cells as the control. **(B)** Alterations of PRMT4 protein abundances and total ADMA-containing protein levels in HPDE6c7, PANC-1, and BxPC-3 cells. Western blotting was performed using β-actin as the internal standard. **(C,D)** Quantitative analysis of PRMT4 abundances and total ADMA-containing proteins levels among HPDE6c7, PANC-1, and BxPC-3 cells detected in **(B)**. Relative protein levels calibrated by β-actin from three biological replicates were presented as mean ± standard deviation and analyzed by the ANOVA method using SPSS 18.0 software. **(E)** The higher viabilities of PANC-1 and BxPC-3 cells compared with HPDE6c7 cells. Cell viabilities were determined by the CCK-8 method. The differences in cell viabilities in three biological replicates were analyzed by the ANOVA method. **(F)** Overexpression of HA-PRMT4 proteins in PANC-1 cells. The stable overexpression of recombinant HA-PRMT4 proteins in PANC-1 cells were validated by western blotting using β-actin as the internal standard. **(G)** The decrease of PANC-1 cell viability induced by PRMT4 overexpression. CCK-8 assay was carried out to measure cell viability of PRMT4-overexpressing PANC-1 cells. PANC-1 cells transfected with packaged lentivirus vectors without ligation of PANC-1 coding sequences were used as the control group. Cell viability differences based on three biological replicates were analyzed by the ANOVA method, **P < 0.01.

However, two other ADMA-containing proteins with lower dimethylation in PANC-1 cells were associated with the acute myeloid leukemia pathway (Figure 5C). Consistently, proteins dimethylated only in HPDE6c7 were enriched in cancer-related pathways such as endometrial cancer, colorectal cancer, and acute myeloid leukemia, while proteins dimethylated only in PANC-1 cells were enriched in the systemic lupus erythematosus pathway which is not directly associated with cancer pathogenesis (Figure 5D). Moreover, these ADMA-containing proteins were also enriched in the Wnt/β-catenin, Hedgehog, tumor growth factor beta (TGF-β), mitogen-activated protein kinase (MAPK), and other cancer-related signaling pathways (Figure 6A). Among them, the dimethylation of TCF, Sos, and AML in PANC-1 cells was significantly lower than the HPDE6c7 cells, while Dvl and PML proteins showed higher dimethylation in PANC-1 cells compared with HPDE6c7 cells (Figure 6A). Four other cancer-related proteins STAT5, TRK, GLI, and CtBP showed no significant dimethylation alterations between HPDE6c7 and PANC-1 cells (Figure 6A). The extensive dimethylation of cancer-related pathways suggested the possible roles of asymmetric arginine dimethylation in PDAC pathogenesis.

### PRMT4 Expression and ADMA-Containing Protein Levels Associated With PDAC Cell Viability

PRMT4 performs essential roles in regulating pancreatic cancer cell functions by catalyzing ADMA formation (Wang Y. P. et al., 2016). In total, 103 ADMA-containing peptides which were identified in our proteomic assay, including 63 peptides differentially methylated in PANC-1 cells, were previously characterized as PRMT4 substrates (Shishkova et al., 2017; Supplementary Tables 1, 2). We also showed that PRMT4 protein abundances in two PDAC cell lines PANC-1 and BxPC-3 cells were significantly lower than the HPDE6c7 cells (Figures 6B,C). Consistently, total ADMA-containing protein levels in PANC-1 and BxPC-3 cells were substantially lower than in the HPDE6c7 cells (Figures 6B,D). Moreover, we found that PANC-1 and BxPC-3 cells showed significantly enhanced cell viabilities compared with HPDE6c7 cells (Figure 6E). To preliminarily analyze the roles of ADMA in PDAC, we overexpressed the PRMT4 gene in PANC-1 cells, which moderately increased asymmetric arginine dimethylation in only two protein bands, possibly due to limited resolution of western blotting (Figure 6F and Supplementary Figure S8). PRMT4 overexpression significantly repressed the viability of PANC-1 cells (Figure 6G). These results provided a basis for elucidating the roles of asymmetric arginine dimethylation in regulating PANC-1 cell functions and PDAC pathogenesis.

## Discussion

Asymmetric arginine dimethylation catalyzed by type I PRMTs like PRMT4 critically regulates cancer development (Blanc and Richard, 2017; Murn and Shi, 2017; Guccione and Richard, 2019; Jarrold and Davies, 2019). As well as the epigenetic regulation mediated by histone methylation, the formation of ADMA in non-histone proteins also emerged as an essential regulator of cancer pathogenesis (Biggar and Shawn, 2015; Guccione and Richard, 2019; Jarrold and Davies, 2019). However, the roles of asymmetric arginine dimethylation in PDAC remains poorly understood, mainly due to the limited ADMA-containing proteins identified in PDAC cells. In the present study, we performed proteomic profiling of ADMA-containing peptides in HPDE6c7 and PANC-1 cells. Most ADMA-containing peptides in PANC-1 cells showed significantly lower dimethylation compared with HPDE6c7 cells. Importantly, for the first time, we showed the significant differences in consensus sequences flanking the ADMA sites between HPDE6c7 and PANC-1 cells. Moreover, ADMA-harboring proteins were associated with RNA splicing and multiple cancer signaling pathways. Finally, we preliminarily validated the roles of asymmetric arginine dimethylation in regulating PANC-1 cell viability by overexpressing PRMT4. These results suggest the essential roles of asymmetric arginine dimethylation in PDAC cells, which provide a basis for the functional elucidation of ADMA-containing proteins in PDAC pathogenesis.

In recent years, immunoaffinity purification using antibodies targeting methylated arginine residues has been successfully applied in the proteomic characterization of protein methylation (Fisk et al., 2013; Guo et al., 2014; Sylvestersen et al., 2014; Geoghegan et al., 2015; Shishkova et al., 2017; Yakubu et al., 2017; Zeeshan et al., 2017), but not in pancreatic cancer. Here, we reported the first global characterization of ADMA-containing proteins in pancreatic cancer, which identified 289 ADMA sites among over 200 proteins by label-free quantitative proteomics combined with affinity purification. Among them, the asymmetric arginine dimethylation of 102 proteins were previously reported, indicative of the reliability of the proteomic dataset. For instance, TAF15, a nuclear RNA-binding protein involved in gene transcription and formation of fusion oncoprotein, was asymmetrically dimethylated at Arg206 to enhance target gene expression (Jobert et al., 2009). Its asymmetric dimethylation at Arg206 was also identified in our proteomic analysis. The methylation of MED12, which sensitizes breast cancer cells to chemotherapy (Wang et al., 2015), was also detected in pancreatic cancer cells. Meanwhile, 99 ADMA sites were newly identified in this study and further investigations might provide new insights into arginine methylation-mediated PDAC development. Our quantitative analysis also showed differential dimethylation of 119 ADMA sites in PANC-1 cells, which needs further experimental validation via calibration by their total protein levels.

As indicated in previous reports, proline and glycine residues were significantly enriched in the flanking sequences of ADMA sites, which were termed as the proline-, glycine-, and methionine-rich (PGM) motif or the glycine and arginine-rich (GAR) motif (Uhlmann et al., 2012; Fisk et al., 2013; Guo et al., 2014; Shishkova et al., 2017). Specifically, glycine residues near ADMA sites were predicted to facilitate the access of arginine residue to PRMT active sites by enhancing conformational flexibility (Blanc and Richard, 2017). Meanwhile, proline residues in the vicinity of ADMA sites might promote ADMA formation by enhancing substrate recognition and increasing hydrophobicity (Shishkova et al., 2017). The significant enrichment of the proline and glycine residues flanking ADMA sites was also observed here in HPDE6c7 and PANC-1 cells, which indicated the unbiased ADMA-containing peptide characterization in this proteomic study. Importantly, we also demonstrated significant enrichment of the leucine residues flanking ADMA sites in pancreatic cells, suggesting the special biochemical mechanism of ADMA formation in pancreatic biology. Considering the hydrophobic property of leucine residues, this observation indicates that higher hydrophobicity might be required for asymmetric arginine dimethylation in pancreatic cells. Furthermore, significant leucine enrichment was observed in ADMA-containing peptides with higher dimethylation or only detected in HPDE6c7 cells, other than those with higher dimethylation or only detected in PANC-1 cells. The biochemical mechanisms and the functional significance of reduced leucine enrichment in pancreatic cancer cells deserves further investigation.

Aberrant RNA splicing contributes to cancer development by inducing transcriptome changes and signaling alterations (Dong and Chen, 2019). Protein arginine methylation also performs key roles in regulating RNA splicing events (Deng et al., 2010; Sanchez et al., 2010; Hu et al., 2017; Jarrold and Davies, 2019). In this methylome study, six spliceosome components were identified in pancreatic cancer cells, further supporting the roles of arginine methylation in regulating RNA splicing during cancer pathogenesis. Moreover, multiple components of cancer signaling pathways were identified in our proteomic assay, such as the Wnt/β-catenin, Hedgehog, TGF-β and MAPK pathways. For instance, transcription factor 7-like 2 (TCF7L2) is one transcriptional partner of the Wnt/β-catenin pathway regulating aerobic glycolysis in pancreatic cancer (Xiang et al., 2018), which showed significantly lower dimethylation in PANC-1 cells in our quantitative proteomics. Moreover, STAT5 protein inactivation mediated Cucurbitacin B-induced G2-M-phase arrest and apoptosis in pancreatic cancer cells (Thoennissen et al., 2009). This study showed that STAT5 protein was also asymmetrically dimethylated in PDAC cells, and its potential roles in pancreatic cancer cell responses to chemotherapies are still worth exploring. In addition, the dimethylated proteins detected only in HPDE6c7 cells were significantly enriched in cancer-related pathways (Figure 5D), which suggested the suppression of their dimethylation in PANC-1 cells. Further investigation is required to broaden knowledge of protein methylation-mediated cancer signaling during PDAC pathogenesis.

PRMT4 performs critical roles in cancer biology by catalyzing asymmetric arginine dimethylation and its expression is repressed in pancreatic cancer cells (Wang Y. P. et al., 2016). The decrease of PRMT4 expression and lower ADMA-containing protein levels were also detected in our study, in two PDAC cell lines compared with the normal pancreatic epithelial cells. Importantly, PRMT4 overexpression caused partial recovery of asymmetric arginine dimethylation and significant suppression of PANC-1 cell viability, which was consistent with a previous report showing the involvement of PRMT4 catalytic activity in regulating PANC-1 cell growth (Wang Y. P. et al., 2016). Moreover, nearly half of the dimethylated proteins identified in HPDE6c7 and PANC-1 cells were previously characterized as PRMT4 substrates by a comprehensive PRMT4 methylome study in breast cancer cells (Shishkova et al., 2017). The ADMA sites identified in this study could serve as clues for further elucidation of the PDAC pathogenic mechanisms mediated by asymmetric arginine dimethylation. In contrast to its high expression in breast cancer (Wang et al., 2014), the greatly repressed expression of PRMT4 in PDAC cells also suggested that the pathogenic roles of PRMT4 in pancreatic cancer might be mediated by tissue-specific signaling mechanisms. In addition, other type I PRMTs such as PRMT1 also critically regulate pancreas development and PDAC pathogenesis (Wang Y. et al., 2016; Lin et al., 2018; Lee et al., 2019; Song et al., 2020), and their pathogenic roles in PDAC cells deserve further investigations.

In summary, we reported the proteomic characterization of ADMA-containing protein profiles between HPDE6c7 and PANC-1 cells in this study. The majority of ADMA-containing proteins showed significantly lowered dimethylation in PANC-1 cells, with a reduced leucine residue enrichment flanking ADMA sites. These ADMA-containing proteins were associated with spliceosome machinery and multiple cancer signaling pathways. Moreover, PRMT4 overexpression partially recovered asymmetric arginine dimethylation and repressed viability in PANC-1 cells. These results provided a comprehensive view of asymmetric arginine dimethylation profiles in PDAC cells, which would facilitate elucidating the pathogenic roles of protein arginine methylation in pancreatic cancer.

## Data Availability Statement

The datasets presented in this study can be found in online repositories. The names of the repository/repositories and accession number(s) can be found in the article/Supplementary Material.

## Author Contributions

JH and TC conceived and designed the study. MW performed the main cellular and proteomic assays, assisted by ZT, YL, YC, and CC. CT, YH, TC, and JH undertook the data analysis and bioinformatics. JH, TC, CT, and WZ wrote the manuscript. All authors read and approved the final manuscript.

## Conflict of Interest

The authors declare that the research was conducted in the absence of any commercial or financial relationships that could be construed as a potential conflict of interest.

## Footnotes

1 http://proteomecentral.proteomexchange.org

2 https://www.maxquant.org/

3 https://www.maxquant.org/perseus/

4 https://plogo.uconn.edu/

5 www.kegg.jp

## Supplementary Material

Supplementary Figure 1 ADMA-containing proteins in the spliceosome machinery. A schematic illustration of ADMA-containing proteins significantly enriched in the spliceosome machinery. The diagram was modified from KEGG pathway database (www.kegg.jp).

Supplementary Figure 2 ADMA-containing proteins in the basal transcription processes. A schematic illustration of ADMA-containing proteins significantly enriched in the basal transcription processes. The diagram was modified from KEGG pathway database (www.kegg.jp).

Supplementary Figure 3 ADMA-containing proteins in the acute myeloid leukemia pathway. A schematic illustration of ADMA-containing proteins significantly enriched in the acute myeloid leukemia pathway. The signaling pathway diagram was modified from KEGG pathway database (www.kegg.jp).

Supplementary Figure 4 ADMA-containing proteins in the chronic myeloid leukemia pathway. A schematic illustration of ADMA-containing proteins significantly enriched in the chronic myeloid leukemia pathway. The signaling pathway diagram was modified from KEGG pathway database (www.kegg.jp).

Supplementary Figure 5 ADMA-containing proteins in the thyroid cancer pathway. A schematic illustration of ADMA-containing proteins significantly enriched in the thyroid cancer pathway. The signaling pathway diagram was modified from KEGG pathway database (www.kegg.jp).

Supplementary Figure 6 ADMA-containing proteins in the Notch signaling pathway. A schematic illustration of ADMA-containing proteins significantly enriched in the Notch signaling pathway. The signaling pathway diagram was modified from KEGG pathway database (www.kegg.jp).

Supplementary Figure 7 ADMA-containing proteins in the systemic lupus erythematosus pathway. A schematic illustration of ADMA-containing proteins significantly enriched in the systemic lupus erythematosus pathway. The signaling pathway diagram was modified from KEGG pathway database (www.kegg.jp).

Supplementary Figure 8 Total ADMA-containing protein levels in PRMT4-overexpressing PANC-1 cells. Total ADMA-containing protein levels were detected by western blotting and protein bands showing the increases of ADMA-containing proteins induced by PRMT4 overexpression were indicated by red arrows.

Supplementary Table 1 A list of total ADMA-containing peptides identified in HPDE6c7 and PANC-1 cells. The protein accessions, names, methylation sites, Andromeda scores, peptide intensities and previous reports of ADMA-containing peptides identified in HPDE6c7 and PANC-1 cells were listed in this table.

Supplementary Table 2 A list of ADMA-containing peptides showing differential dimethylation between HPDE6c7 and PANC-1 cells. Information of ADMA-containing peptides with significantly differential dimethylation between HPDE6c7 and PANC-1 cells were analyzed by the Perseus software and listed in this table, including protein accessions, names, sequence windows, logarithm-transformed intensities and *P*-values from the Student *T*-tests.
