# Supplementary figures and images for: Proteome-Wide Alterations of Asymmetric Arginine Dimethylation Associated With Pancreatic Ductal Adenocarcinoma Pathogenesis
Source: Front Cell Dev Biol. 2020 Dec 3;8:545934. doi: 10.3389/fcell.2020.545934 (PMC7744470; doi:10.3389/fcell.2020.545934)

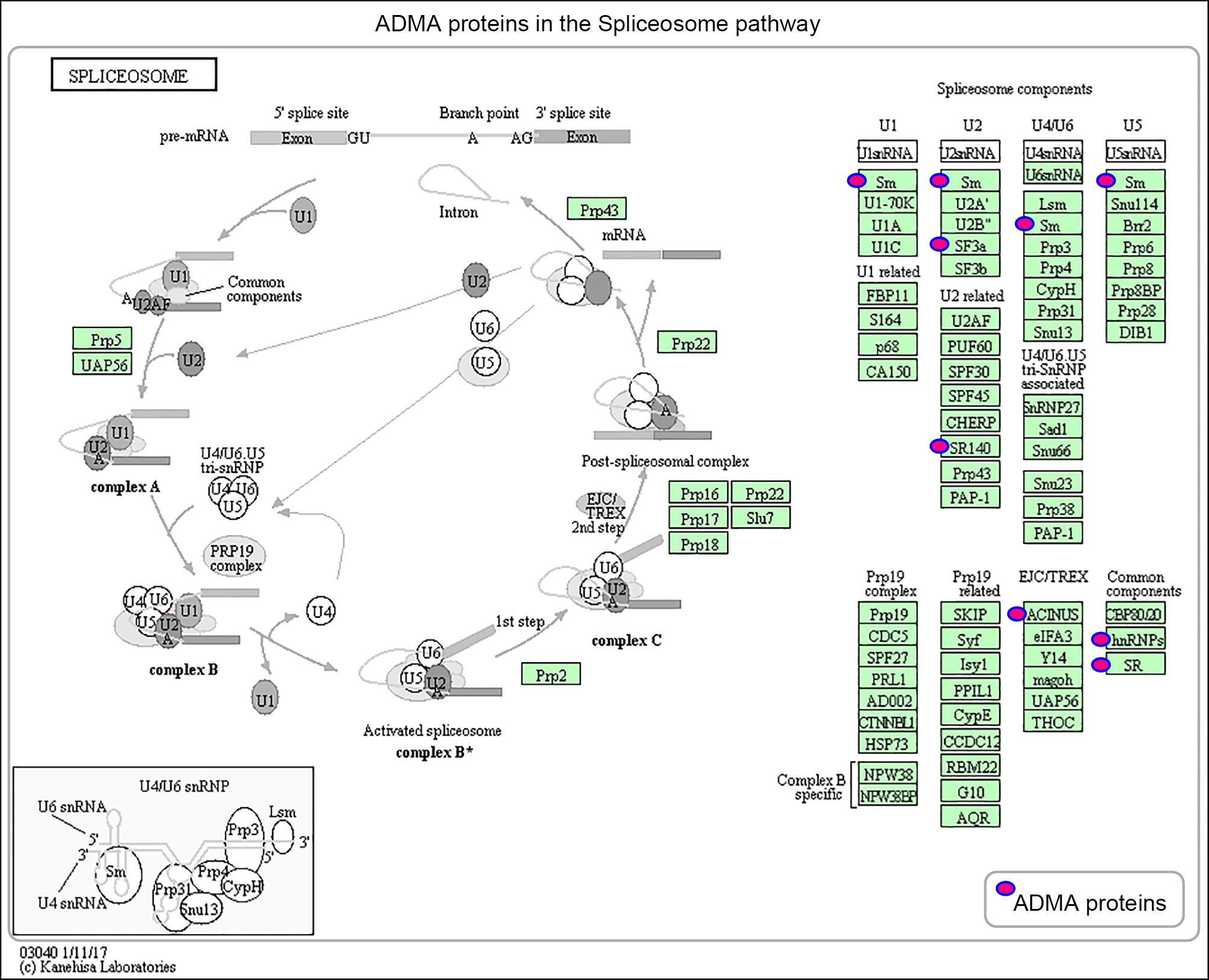

Supplement: Supplementary Figure 1 — ADMA-containing proteins in the spliceosome machinery. A schematic illustration of ADMA-containing proteins significantly enriched in the spliceosome machinery. The diagram was modified from KEGG pathway database (www.kegg.jp). [file Image_1.TIF]

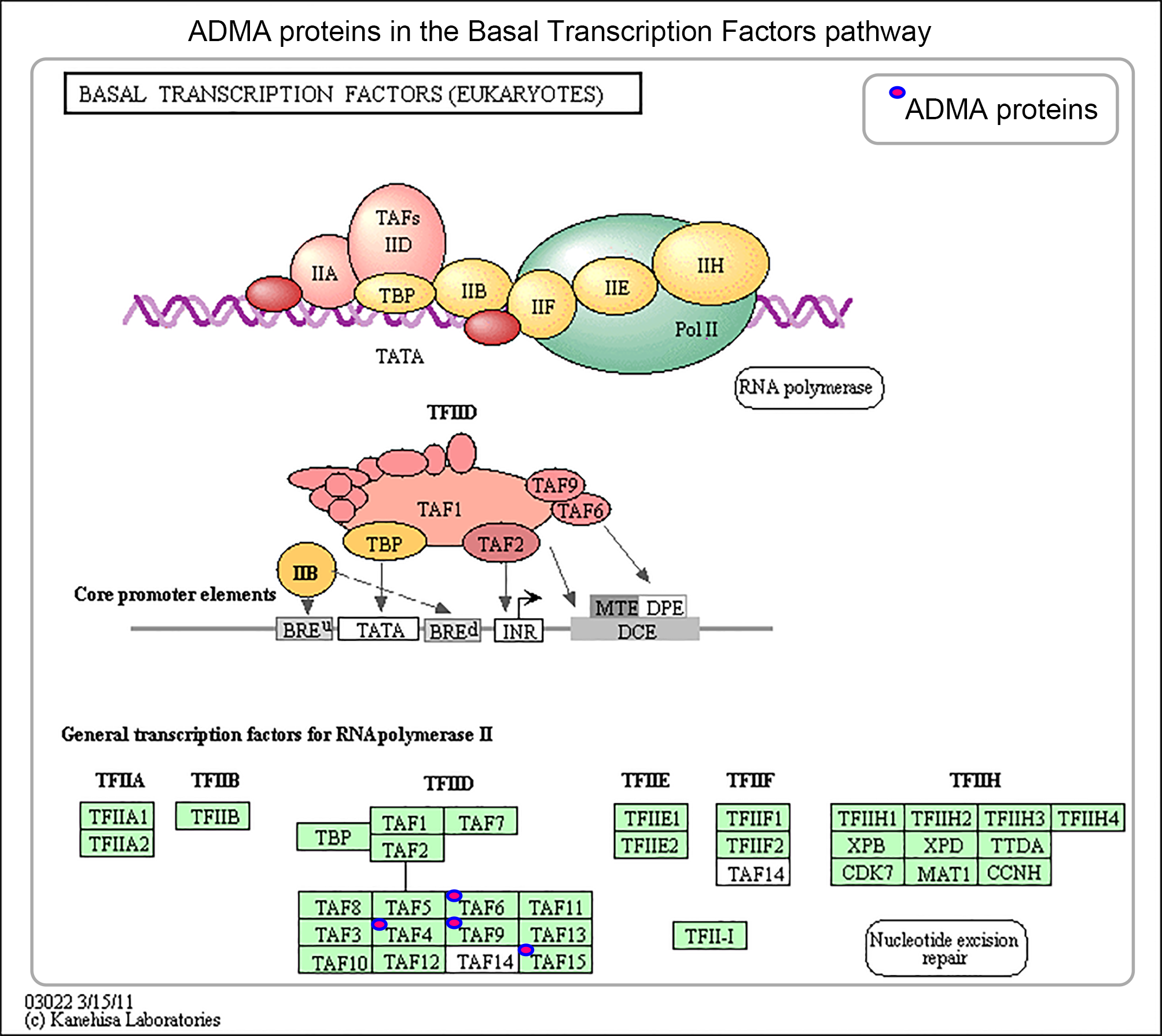

Supplement: Supplementary Figure 2 — ADMA-containing proteins in the basal transcription processes. A schematic illustration of ADMA-containing proteins significantly enriched in the basal transcription processes. The diagram was modified from KEGG pathway database (www.kegg.jp). [file Image_2.TIF]

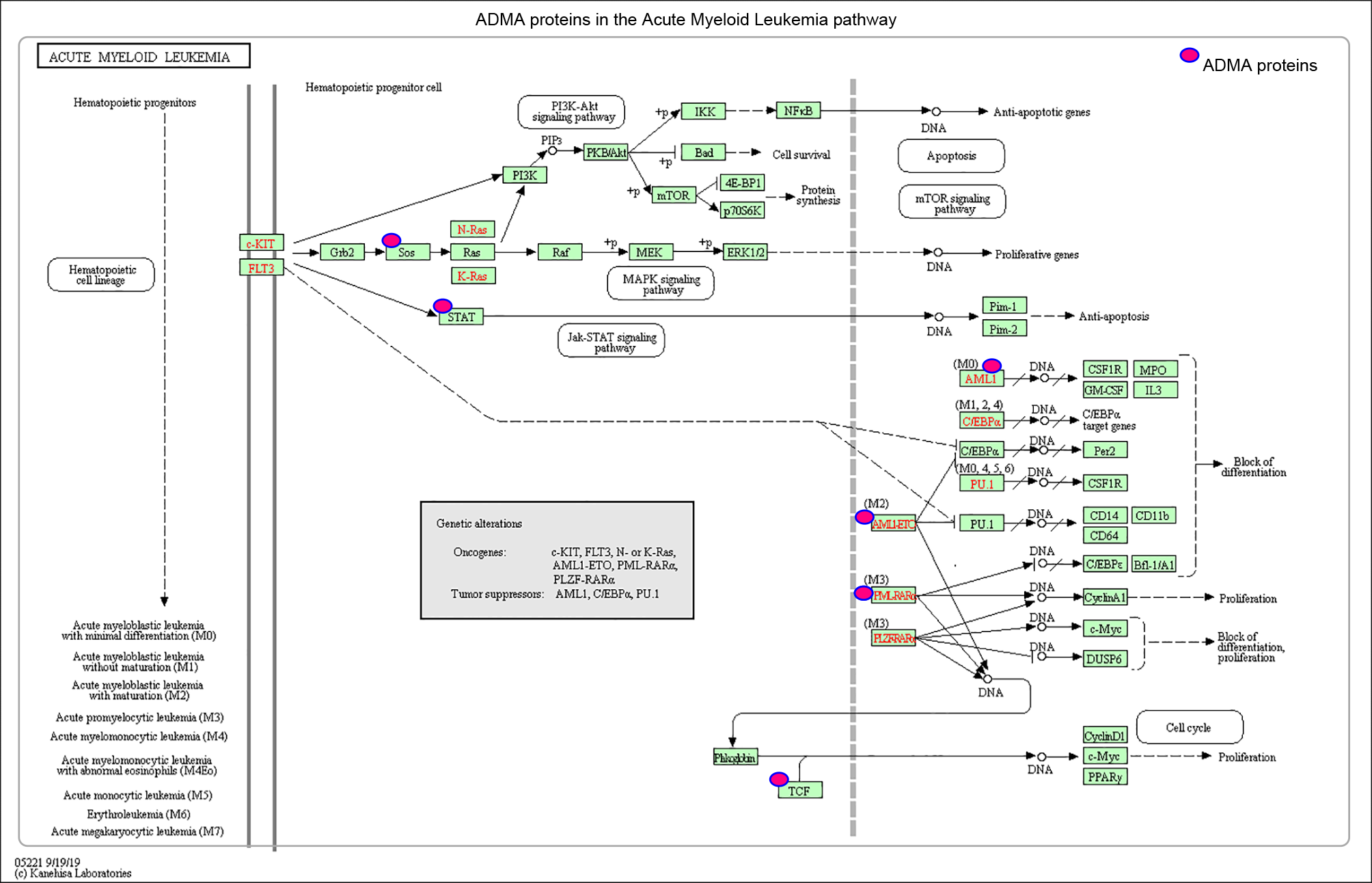

Supplement: Supplementary Figure 3 — ADMA-containing proteins in the acute myeloid leukemia pathway. A schematic illustration of ADMA-containing proteins significantly enriched in the acute myeloid leukemia pathway. The signaling pathway diagram was modified from KEGG pathway database (www.kegg.jp). [file Image_3.TIF]

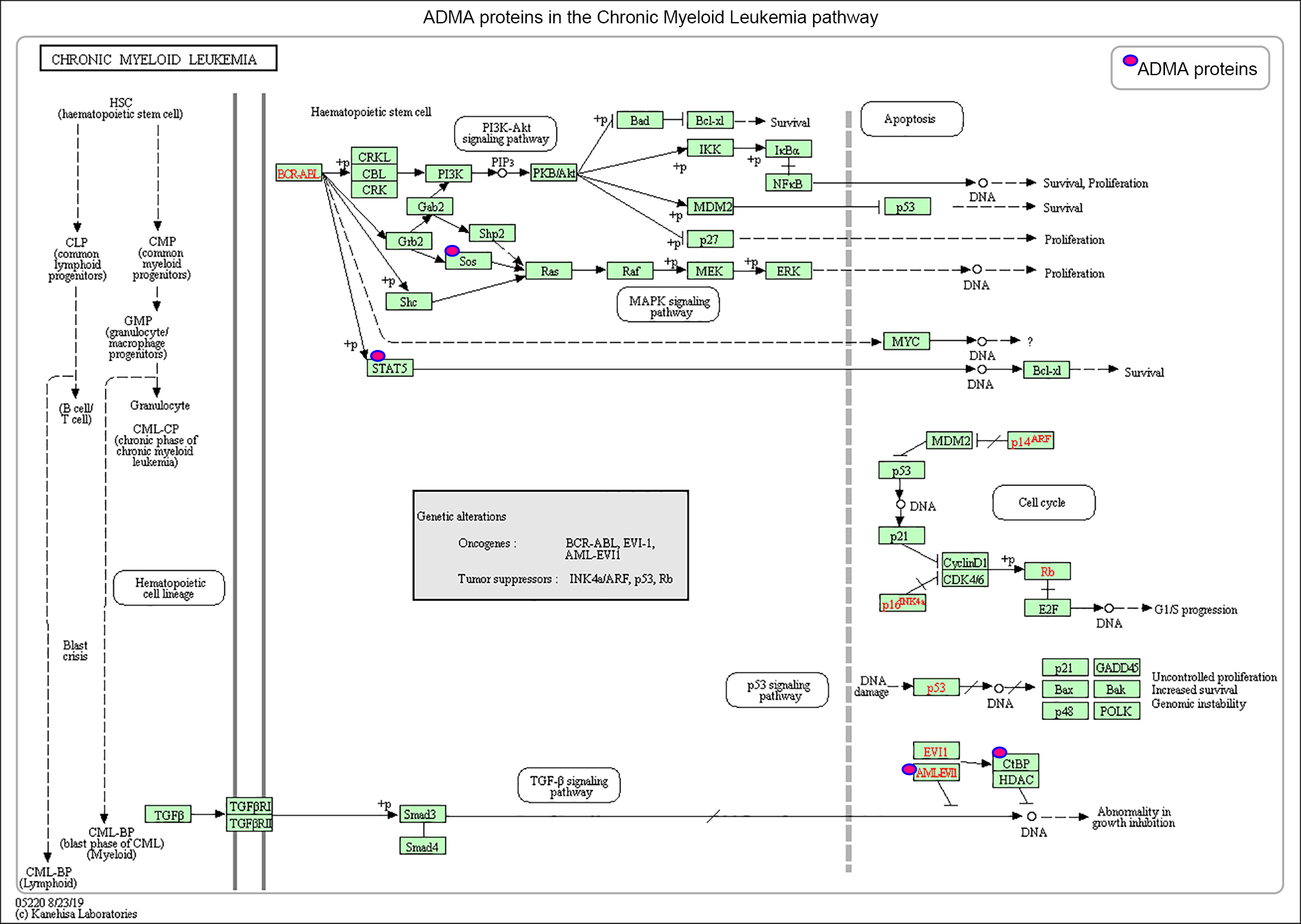

Supplement: Supplementary Figure 4 — ADMA-containing proteins in the chronic myeloid leukemia pathway. A schematic illustration of ADMA-containing proteins significantly enriched in the chronic myeloid leukemia pathway. The signaling pathway diagram was modified from KEGG pathway database (www.kegg.jp). [file Image_4.TIF]

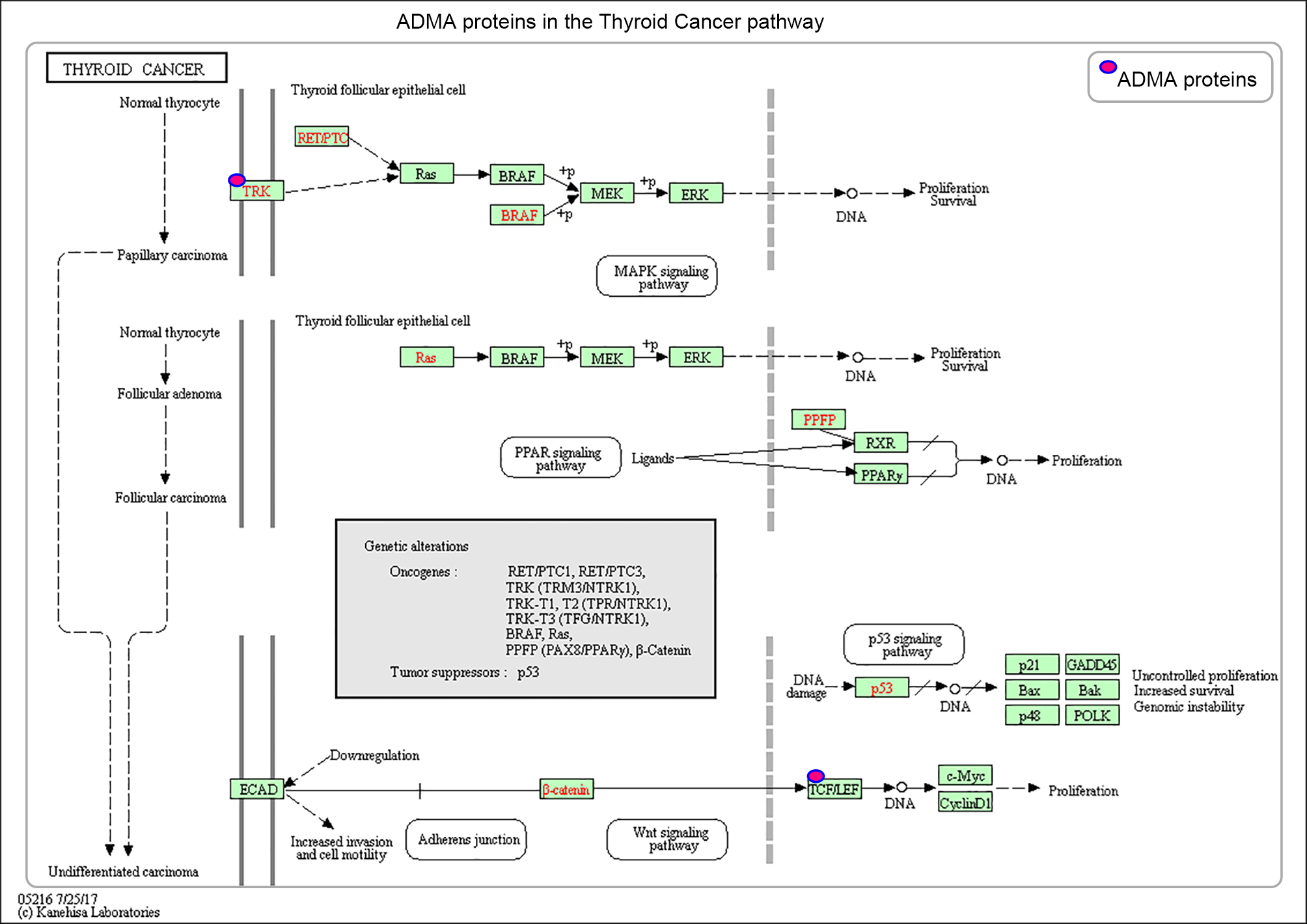

Supplement: Supplementary Figure 5 — ADMA-containing proteins in the thyroid cancer pathway. A schematic illustration of ADMA-containing proteins significantly enriched in the thyroid cancer pathway. The signaling pathway diagram was modified from KEGG pathway database (www.kegg.jp). [file Image_5.TIF]

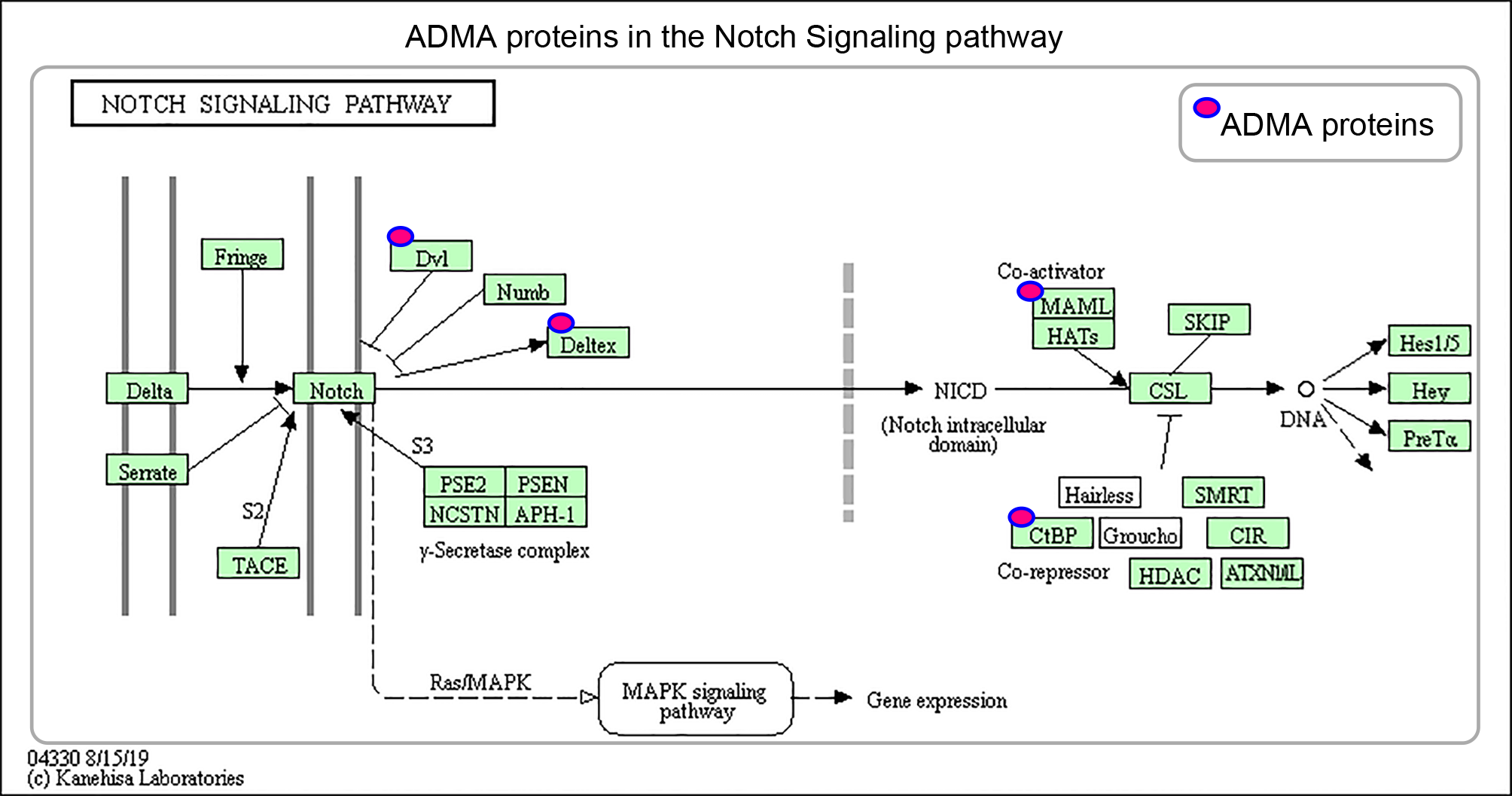

Supplement: Supplementary Figure 6 — ADMA-containing proteins in the Notch signaling pathway. A schematic illustration of ADMA-containing proteins significantly enriched in the Notch signaling pathway. The signaling pathway diagram was modified from KEGG pathway database (www.kegg.jp). [file Image_6.TIF]

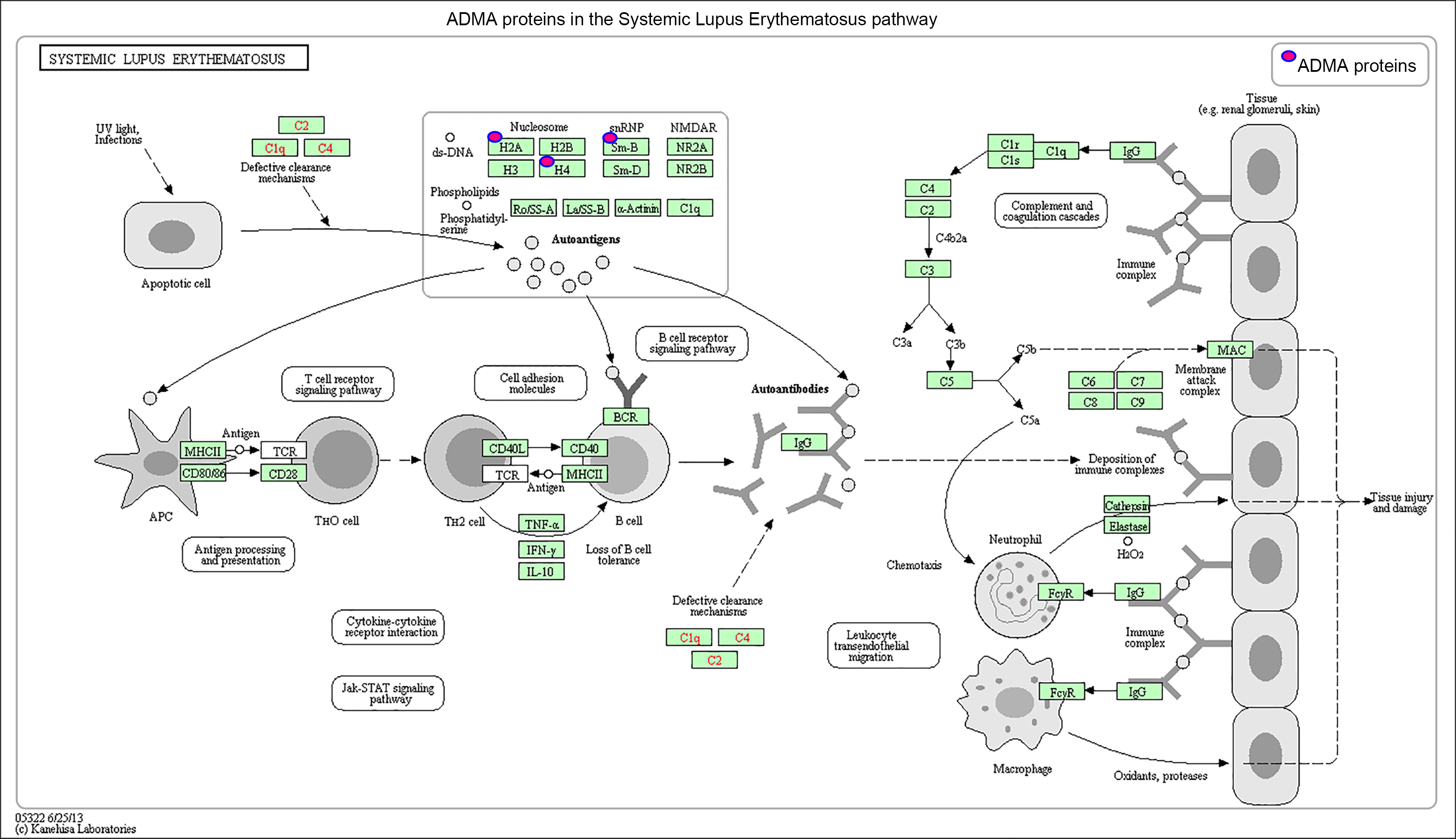

Supplement: Supplementary Figure 7 — ADMA-containing proteins in the systemic lupus erythematosus pathway. A schematic illustration of ADMA-containing proteins significantly enriched in the systemic lupus erythematosus pathway. The signaling pathway diagram was modified from KEGG pathway database (www.kegg.jp). [file Image_7.TIF]

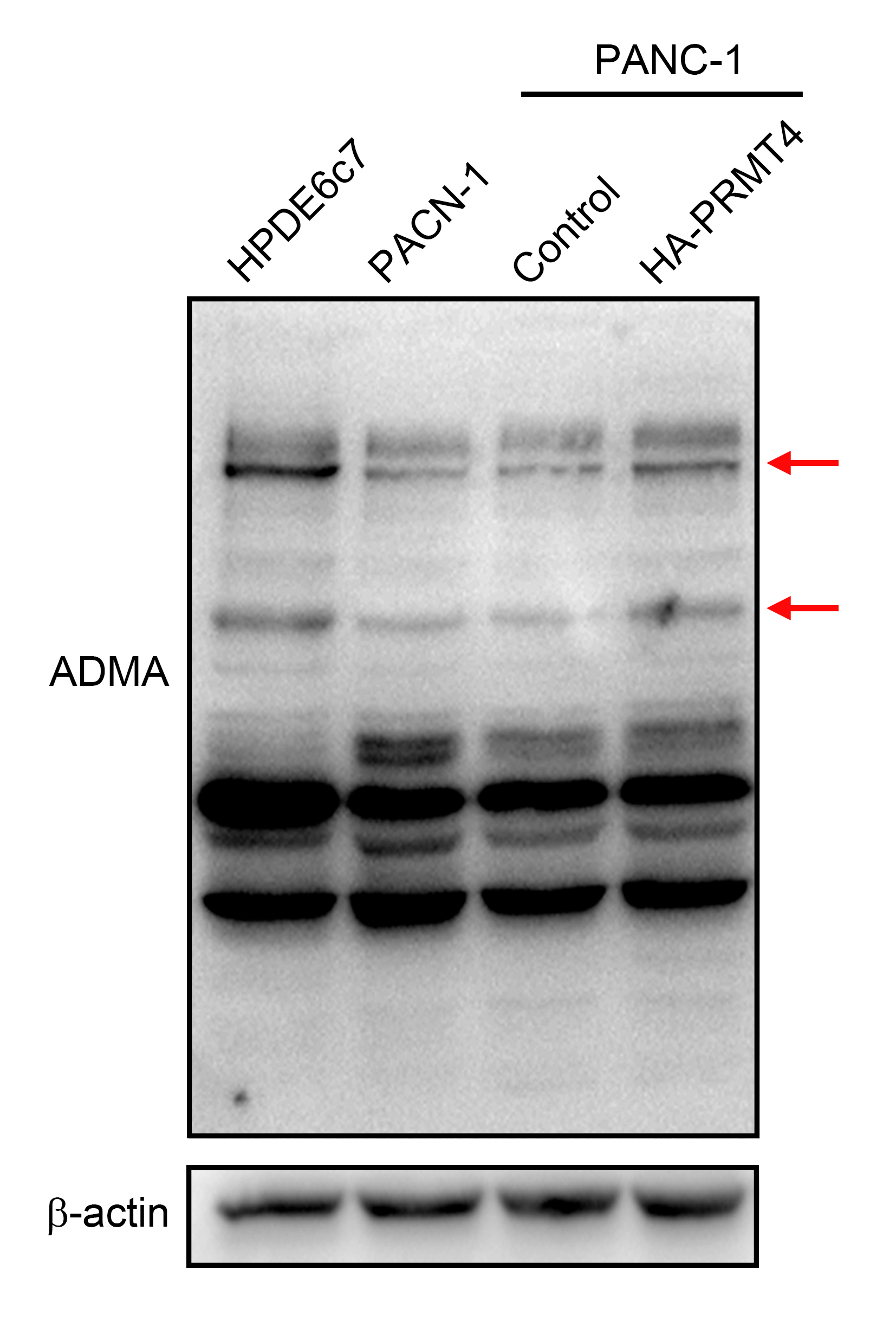

Supplement: Supplementary Figure 8 — Total ADMA-containing protein levels in PRMT4-overexpressing PANC-1 cells. Total ADMA-containing protein levels were detected by western blotting and protein bands showing the increases of ADMA-containing proteins induced by PRMT4 overexpression were indicated by red arrows. [file Image_8.JPEG]
